# Impact of molybdenum out diffusion and interface quality on the performance of sputter grown CZTS based solar cells

**DOI:** 10.1038/s41598-017-01605-7

**Published:** 2017-05-02

**Authors:** Goutam Kumar Dalapati, Siarhei Zhuk, Saeid Masudy-Panah, Ajay Kushwaha, Hwee Leng Seng, Vijila Chellappan, Vignesh Suresh, Zhenghua Su, Sudip Kumar Batabyal, Cheng Cheh Tan, Asim Guchhait, Lydia Helena Wong, Terence Kin Shun Wong, Sudhiranjan Tripathy

**Affiliations:** 1 Institute of Materials Research and Engineering, A*STAR (Agency for Science, Technology and Research), 2 Fusionopolis Way, Innovis, #08-03, Singapore, 138634 Singapore; 2 NOVITAS, School of Electrical and Electronic Engineering, Block S2, Nanyang Technological University, Nanyang Avenue, Singapore, 639798 Singapore; 3 Department of Metallurgy Engineering and Materials Science, Indian Institute of Technology Indore, Indore, MP 453552 India; 4 Energy Research Institute @ NTU, Nanyang Technological University, 50 Nanyang Drive, Research Techno Plaza, X-Frontier Block, Level 5, Singapore, 637553 Singapore; 5 Amrita Centre for Industrial Research and Innovation (ACIRI), Amrita School of engineering, Coimbatore, Amrita University, Tamil Nadu, 641112 India; 6 School of Materials Science and Engineering, Nanyang Technological University, 50 Nanyang Avenue, Singapore, 639798 Singapore

## Abstract

We have investigated the impact of Cu_2_ZnSnS_4_-Molybdenum (Mo) interface quality on the performance of sputter-grown Cu_2_ZnSnS_4_ (CZTS) solar cell. Thin film CZTS was deposited by sputter deposition technique using stoichiometry quaternary CZTS target. Formation of molybdenum sulphide (MoS_x_) interfacial layer is observed in sputter grown CZTS films after sulphurization. Thickness of MoS_x_ layer is found ~142 nm when CZTS layer (550 nm thick) is sulphurized at 600 °C. Thickness of MoS_x_ layer significantly increased to ~240 nm in case of thicker CZTS layer (650 nm) under similar sulphurization condition. We also observe that high temperature (600 °C) annealing suppress the elemental impurities (Cu, Zn, Sn) at interfacial layer. The amount of out-diffused Mo significantly varies with the change in sulphurization temperature. The out-diffused Mo into CZTS layer and reconstructed interfacial layer remarkably decreases series resistance and increases shunt resistance of the solar cell. The overall efficiency of the solar cell is improved by nearly five times when 600 °C sulphurized CZTS layer is applied in place of 500 °C sulphurized layer. Molybdenum and sulphur diffusion reconstruct the interface layer during heat treatment and play the major role in charge carrier dynamics of a photovoltaic device.

## Introduction

Cu_2_ZnSnS_4_ (CZTS) is extensively studied as an active absorber layer in solar cell architecture as it is non-toxic, earth-abundant and has the potential to demonstrate excellent solar cell performance^1–7^. However, presence of impurities, inter-diffusion of elemental atoms, interfacial layer formation, secondary phase formation and non-stoichiometry limits the solar cell efficiency^8–12^. To address the issues, we demonstrate the impact of Mo out-diffusion and interface layer quality on the performance of sputter-grown CZTS based solar cells.

The structural, crystallographic, and electrical properties of the CZTS layer can be tailored by optimizing stoichiometry-compositions, which can significantly control the formation of native defects^13–16^. The chemical composition and structural properties of the CZTS layer depend on the deposition process and sulphurization temperature^17, 18^. Among several deposition methods^9–14, 19, 20^, sputter deposition technique provides precise control of film thickness over large area, *in-situ* crystal quality by tuning working pressure during deposition, and chemical composition of the deposited layer^21–23^. Furthermore, deposition of CZTS using a single-step sputtering from a quaternary Cu_2_ZnSnS_4_ target offers many advantages, including the uniform composition of the thin film, smooth surface, simple process and high reproducibility^24^.

Electrical properties of CZTS absorber are significantly improved after thermal annealing, mostly performed in sulphur-rich atmosphere^25, 26^. During thermal annealing of CZTS, secondary phase formation, inter-diffusion of elemental atoms, and growth of interfacial layer between CZTS and Mo are generally observed^27^. The sulphurization temperature plays the crucial role on the device performance. Chalapathy *et al*. have grown high-quality CZTS film when sputtered Cu/ZnSn/Cu metal layer were sulphurized at 560–580 °C for 30 min^28^. The formation of bilayer morphology is reported when sulphurization temperature was 560 °C in comparison to 580 °C^28^. Sulphurization temperature has a strong influence on the microstructural properties and diffusion of elements in the films^28–31^. Even though there are several reports available on the sulphurization of sputter-grown CZTS, the effect of sulphurization temperature on interface quality of sputter-grown CZTS using quaternary Cu_2_ZnSnS_4_ target is not investigated. Since sulphur is present in the quaternary Cu_2_ZnSnS_4_ target; interfacial layer formation mechanism is different than the CZTS layer grown by sputter with multilayer layer metal films followed by sulphur annealing. CZTS-Mo interface plays a critical role to decide the efficiency of the solar cells^11, 12^ and it is difficult to avoid the formation of MoS_x_ layer. Therefore, in this work, the impact of Mo out-diffusion into CZTS films and formation of MoS_x_ layer is addressed and solar cell performance is evaluated for the sputter-grown CZTS film. The impact of elemental metal impurity in the interface layer has also been addressed.

## Experimental

CZTS thin films were deposited on Mo-coated soda-lime glass using RF-magnetron sputtering at room temperature. The Mo-coated soda-lime glass substrates were ultrasonicated in isopropyl alcohol (IPA) for 10 minutes and dried with nitrogen gas flow. Then, the substrates were immediately loaded into the sputtering chamber. Sputter deposition was carried out using a stoichiometric Cu_2_ZnSnS_4_ target with pure Ar gas as the sputtering gas with a base pressure of 10^−7^ Torr. The CZTS thin film was deposited with RF power of 150 W at a working pressure of 3.3 mTorr. The sputter-deposited thin films were sealed in a quartz tube together with sulphur powder following which they were annealed in a furnace at 500–600 °C for 10 minutes. The ramping rate during annealing was 19.3 °C/min. Two different thicknesses of CZTS films 550 nm and 650 nm were investigated. Scanning electron microscopy (SEM) was used to investigate the structural quality and interface layer thickness of sputter deposited CZTS thin film. Crystal quality and secondary phase formation were characterized by X-ray diffraction (XRD) and Raman spectroscopy. X-ray diffraction analysis was performed using a Bruker D8 general area detector XRD system (GADDS) in a θ–2θ scan using CuKα (λ = 0.154 18 nm) radiation. The Raman spectra were collected using a JY LABRAM micro-Raman system with 488 nm visible Raman excitation. Secondary ion mass spectrometry (SIMS) was employed to study the elemental distribution of the CZTS thin film after thermal treatment.

A CdS buffer layer with ~ 60 nm thickness was deposited on sulphurized CZTS film by chemical bath deposition (CBD). Then, 50 nm i-ZnO followed by ~600 nm ZnO:Al layers were deposited by RF and DC magnetron sputtering, respectively. Finally, conductive silver paste was printed on AZO layer to form top contact fingers. Solar cell performance was investigated through current density-voltage (J-V) characteristics using Xe-based solar simulator (VS-0852 and KEITHLEY 2612 A) to provide simulated 1 sun AM 1.5 G illumination with the intensity of 100 mW/cm^2^. The light source was calibrated with a standard Si reference cell. The total area of the cell was 0.16 cm^2^. External quantum efficiency (EQE) was measured by PVE300 (Bentham) IPCE Instrument equipped with a xenon/quartz halogen light source and calibrated with Si/Ge reference detectors. The transient photovoltage (TPV) measurements were carried on the p-CZTS/n-CdS heterojunction solar cell to determine the photo-generated carrier lifetime using a pulsed Nd:YAG laser (pulse width <5 ns) of wavelength 532 nm with the device illuminated with strong white light as background. TPV signals were recorded using a digital oscilloscope (Agilent, 1 GHz) for different white light conditions. The transient signals were fitted with a single exponential function in order to estimate the charge carrier lifetime.

## Results and Discussion

**Figure 1:**
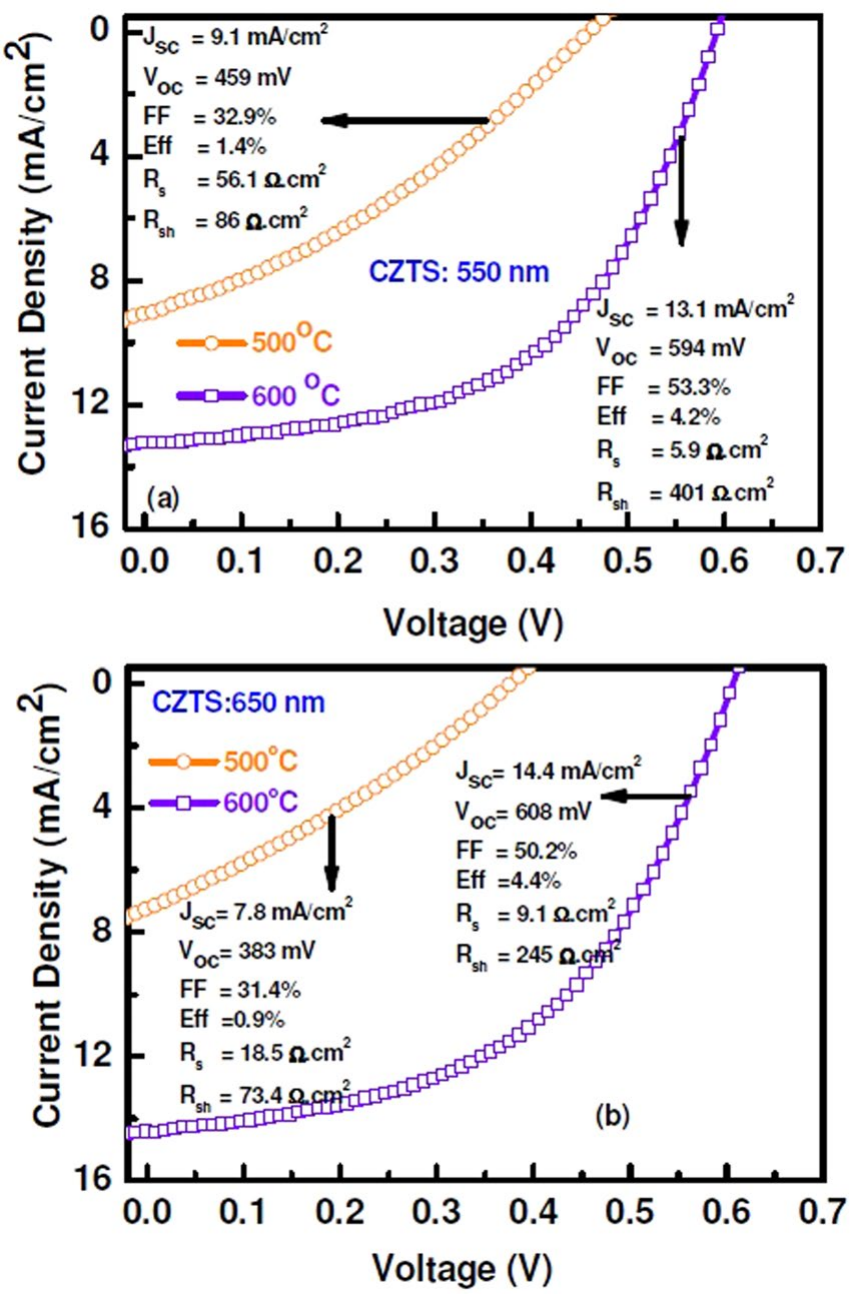
Current-voltage characteristics of CZTS solar cells with CZTS thickness of (**a**) 550 nm and (**b**) 650 nm. CZTS layer are sulphurized at 500 °C and 600 °C.

Current-voltage characteristic of the CZTS thin film solar cell is presented in Fig. 1. The solar cells were fabricated with two different absorber layer thicknesses of 550 nm and 650 nm. The solar cell with 550 nm thick CZTS film (sulphurized at 500 °C) showed short-circuit current density (*J*_*sc*_) and the open-circuit voltage (*V*_*oc*_) of 10 mA/cm^2^ and 450 mV, respectively (Fig. 1a). The values of *J*_*sc*_ and *V*_*oc*_ significantly enhanced to 13.2 mA/cm^2^ and 600 mV, when CZTS layer with the same thickness (550 nm) was sulphurized at 600 °C. The overall efficiency of the solar cell is increased from 1.4% to ~4.2%, when sulphurization temperature of CZTS changed from 500 °C and 600 °C. Moreover, the *J*_*sc*_ and *V*_*oc*_ slightly enhance when absorber layer thickness increased to 650 nm (Fig. 1(b)). The overall efficiency of the solar cell is increased to ~4.4%.

**Figure 2:**
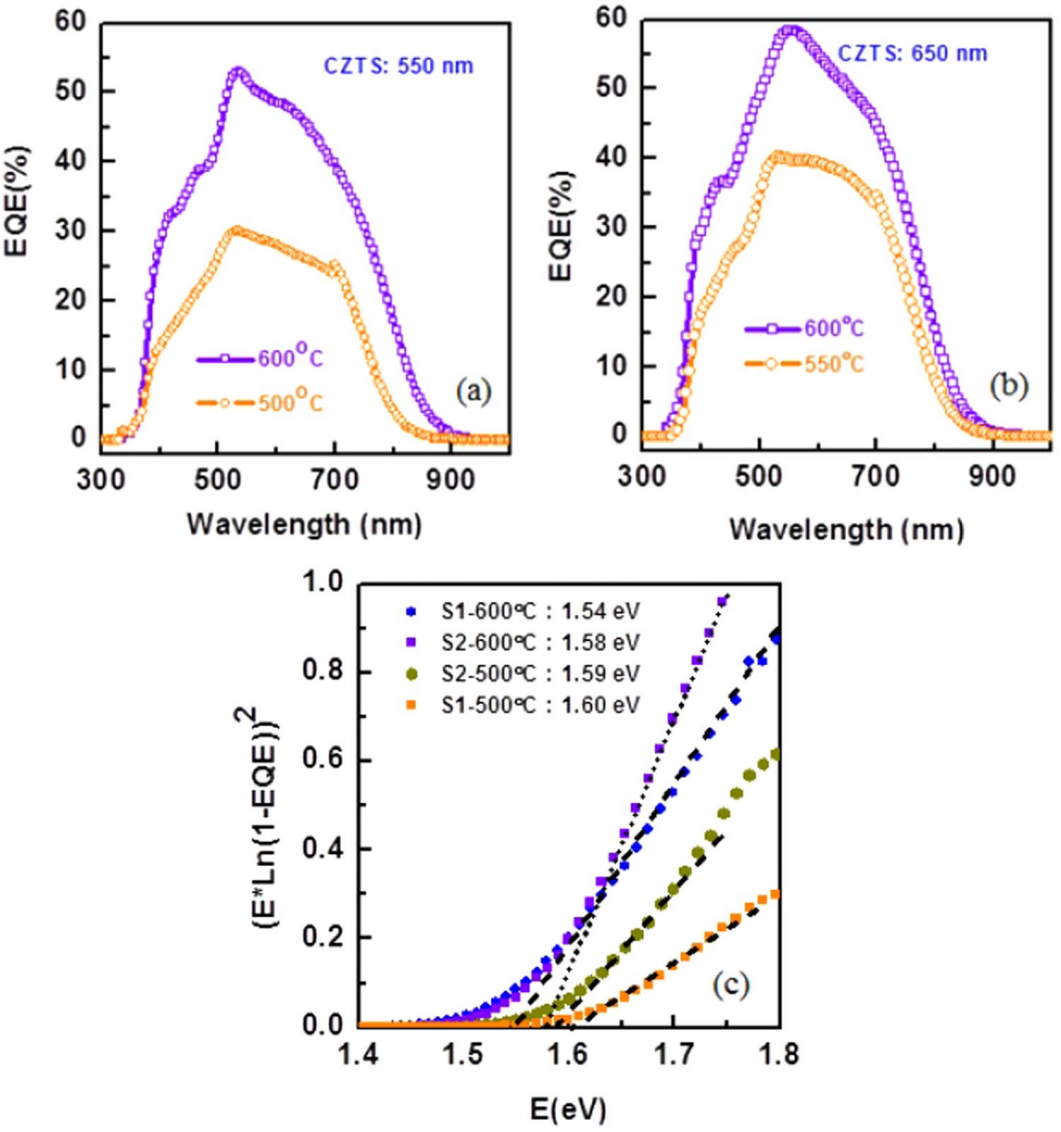
EQE spectra of CZTS solar cells with CZTS thickness of (**a**) 550 nm and (**b**) 650 nm after CZTS layer sulphurized at 500 °C and 600 °C. (**c**) Variation of [E.ln (1-EQE)]^2^ versus E to extract bandgap of the CZTS layer.

External quantum efficiency (EQE) of CZTS solar cell is also evaluated (Fig. 2). The CZTS layer sulphurized at 600 °C showed significantly higher EQE in the wavelength region from 400 nm to 900 nm for both the thicknesses suggesting the reduction of recombination centers in the bulk CZTS absorber layer. EQE data was also used to estimate the electronic band gap (E_g_) of the CZTS absorber by plotting the [E × In(1-EQE)]^2^ versus energy, E^30^. The optical bandgap, E_g_, is estimated from the intercept of E axis with extrapolated linear segments of the [E.ln (1-EQE)]^2^ curve (Fig. 2c). The estimated optical bandgap of CZTS is varying in between 1.54 eV −1.6 eV. The optical band gap of the CZTS layer with different thicknesses is almost similar.

**Figure 3:**
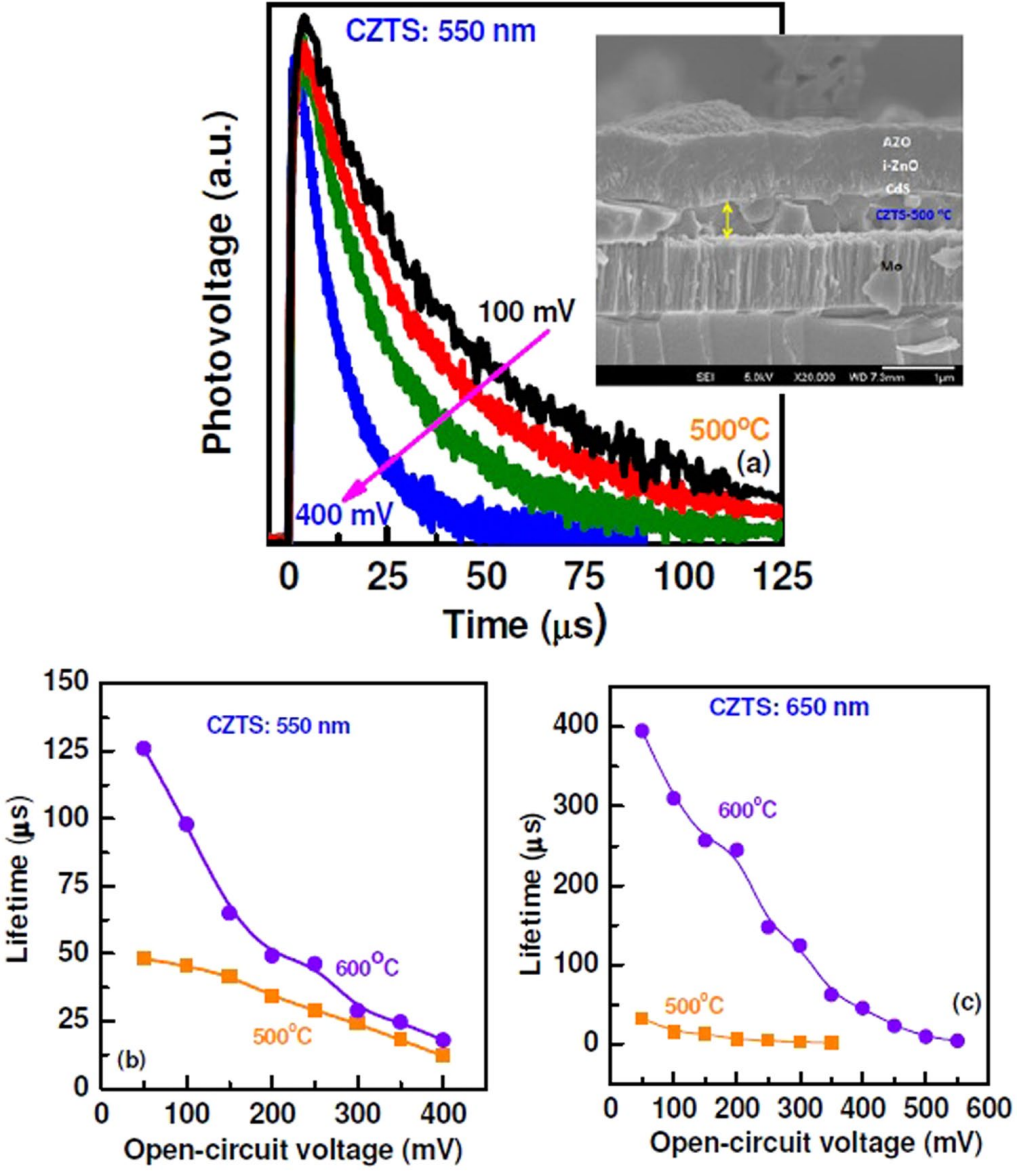
(**a**) Transient photovoltage characteristics of CZTS/CdS junction with 550 nm thick CZTS layer sulphurized at 500 °C. Carrier life time characteristics of the solar cell with CZTS thickness of (**b**) 550 nm and (**c**) 650 nm after sulphurized at 500 °C and 600 °C.

Transient photovoltage (TPV) measurements were performed to investigate the electronic properties of sputtered grown-CZTS based solar cells. Figure 3 shows TPV characteristics of the CZTS solar cell and estimated carrier lifetime plots. TPV characteristics of solar cells with 550 nm thick CZTS layer (sulphurized at 500 °C) are given in Fig. 3a. The exponential decay of the photo-voltage with respect to time directly represents the faster recombination of the photo-generated charge carriers. A sequential slower recombination is observed at lower open circuit potential. The lifetime of photo-generated charge carriers is calculated from TPV profile as presented in Fig. 3b–c and compared with the solar cell in which CZTS layer is sulphurized at the different temperature. The lifetime (125 µs) of photo-generated charge carrier is found 2.5 times higher in the film sulphurized at 600 °C than at 500 °C. Such increase in lifetime of photo-generated charge carriers indicates lower recombination rate; which only is possible when there is a significant improvement in the film quality at this temperature. In the case of 650 nm thick CZTS film, carrier lifetime is further increased, when compared to 550 nm thick film (Fig. 3c). Even though thicker device (650 nm sulphurized at 600 °C) shows significantly higher carrier lifetime and EQE, the power conversion efficiency of the device is similar to the device fabricated with thinner CZTS layer (550 nm). There is a considerable difference in solar cell parameters. In fact, series resistance and shunt resistance significantly varies with the thickness of CZTS layer.

The series and shunt resistance of all four devices are calculated (Fig. 1). The resistance values change significantly with sulphurization temperature and film thickness. The ten times lower series resistance and five times higher shunt-resistance are measured, when the solar cell is fabricated with CZTS layer of thickness 550 nm and sulphurized at 600 °C as compared to 500 °C. Since shunt resistance mainly originates from the recombination at defect states, the increase in the shunt resistance indicates the reduction of defect states. The series resistance and shunt resistance of the device with CZTS thickness of 650 nm are significantly different compared with the device of CZTS thickness 550 nm. It is important to note that the device fabrication process is similar and the device was fabricated after thermal treatment of CZTS layer. Thus, any variation in the resistance can be safely assumed to have originated due to the rear surface recombination. This suggests that the interface quality between CZTS and Mo plays an important role in the overall carrier collection efficiency and this layer is significantly dependent on the thickness of sputter-grown CZTS layer. The interface layer formation is particularly important for sputter CZTS film using quaternary Cu_2_ZnSnS_4_ target, as sulphur is present in the sputter film.

**Figure 4:**
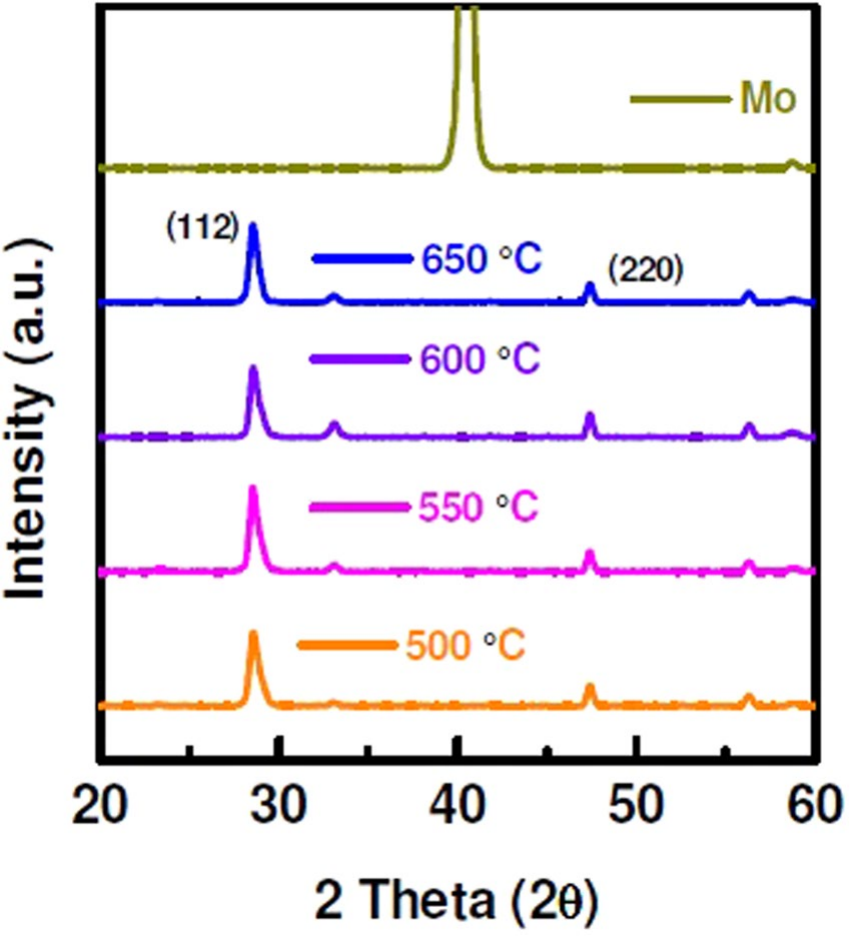
XRD pattern of CZTS layer after annealed at different temperatures.

Solar cell characterization data shows that sulphurization of CZTS film at 600 °C has better performance irrespective of layer thickness. Furthermore, shunt and sheet resistances indicate that annealing at 600 °C is more beneficial. Because at this temperature material quality of CZTS film as well as interface properties improve significantly. However, it is worth mentioning that the CZTS solar cells with thicker CZTS layer suffer from high series resistance and low shunt resistance. Therefore, structural properties of CZTS films are investigated to identify the cause for such difference in solar cell performance. XRD analysis shows all films exhibit a major diffraction peak at 28.5° corresponding to (112) planes of kesterite CZTS phase^28–30^, for as-deposited films and after thermal treatment, as shown in Fig. 4. There is no significant structural change observed with the change in annealing temperature for sputter grown CZTS on Mo-coated substrate. The secondary phase formation for the film sulphurized at 500 °C and 600 °C is similar in XRD measurements. The full width half maximum (FWHM) values of (112) and (220) peaks of annealed CZTS films (at different temperatures) are tabulated in Table 1. The corresponding crystal size of the main XRD peaks of CZTS(112) and CZTS(220) of samples annealed at different temperatures was also determined from the Scherrer formula, D = K.λ/β.Cosθ, where D, k, λ, β and θ are grain size, dimensionless shape factor, X-ray wavelength, line broadening at FWHM in radians and Bragg angle, respectively. The crystal size of sputter-grown CZTS is ~11.3 nm after thermal treatment at 500 °C. By increasing annealing temperature, crystal sizes increases slightly, mainly originated from the improvement of the crystal quality of the films.

**Table 1:** Comparison of FWHM and crystal size of sputter grown CZTS with annealing temperature.

Annealing temperature	FWHM (degree) At (112)	Crystal size (nm)	FWHM (degree) At (220)	Crystal size (nm)
500 °C	0.72	11.3	0.36	22.7
550 °C	0.68	12.4	0.34	24.1
600 °C	0.66	12.8	0.30	27.3
650 °C	0.64	13.3	0.28	29.4

**Figure 5:**
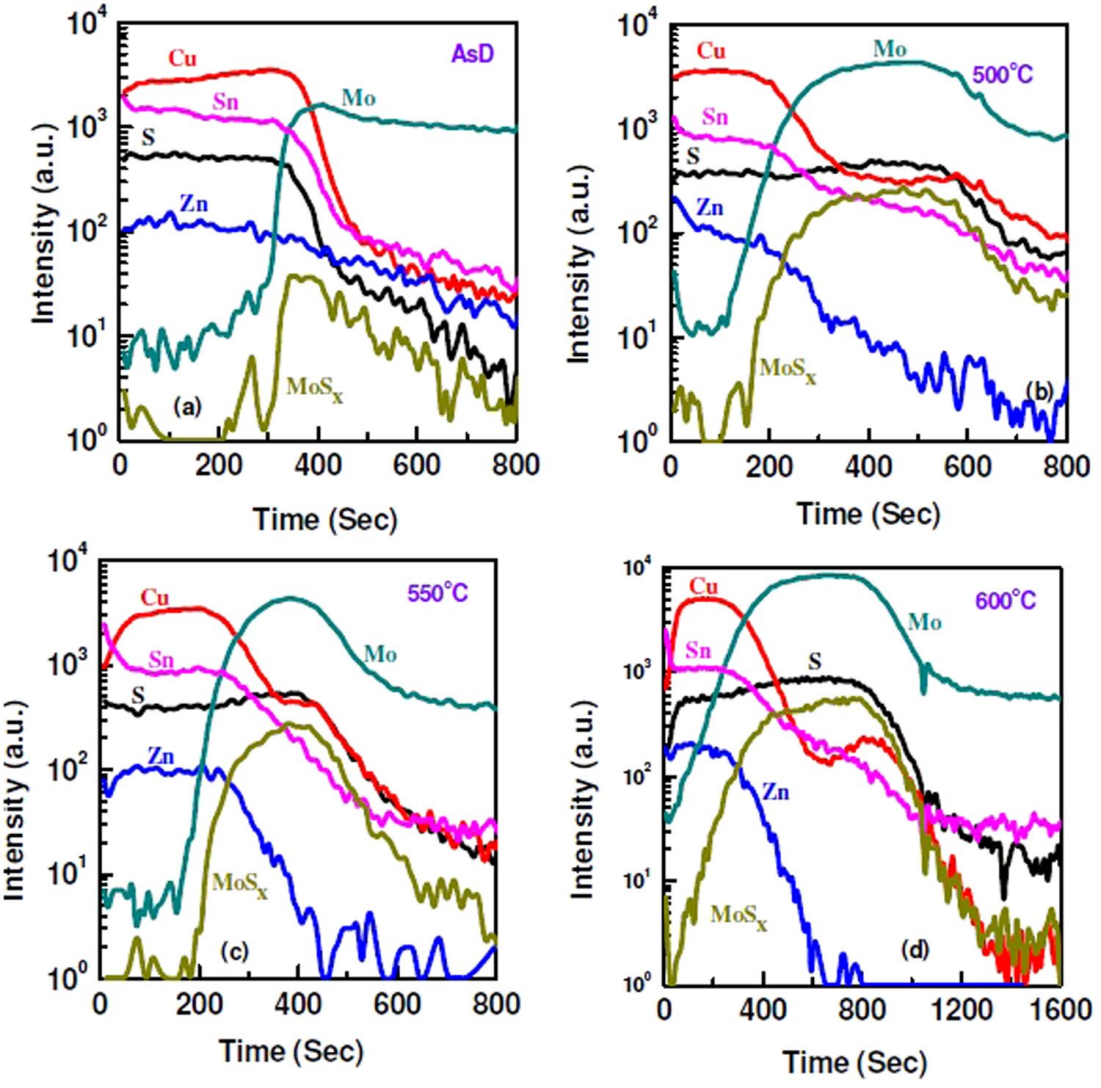
SIMS profile of elemental atoms from CZTS/Mo structure for (**a**) as-deposited CZTS film and after sulphurizationat (**b**) 500 °C, (**c**) 550 °C and (**d**) 600 °C.

**Figure 6:**
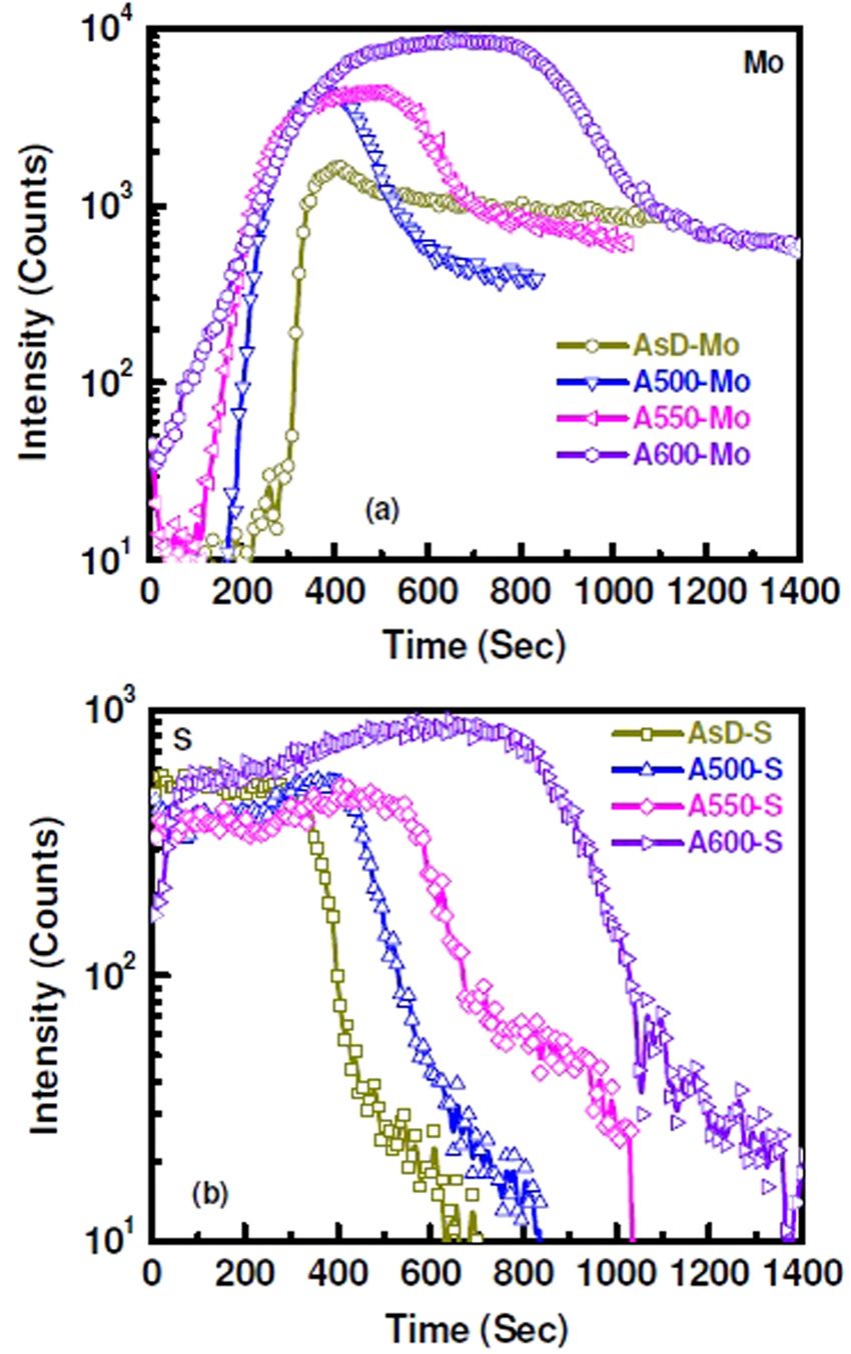
Comparison of (**a**) Mo and (**b**) S distribution with varying annealing temperature. “AsD” denotes as deposited sample and “A500”, “A550”, “A600” denotes samples annealed at 500 ^ο^C, 550 ^ο^C, and 600 ^ο^C respectively.

**Figure 7:**
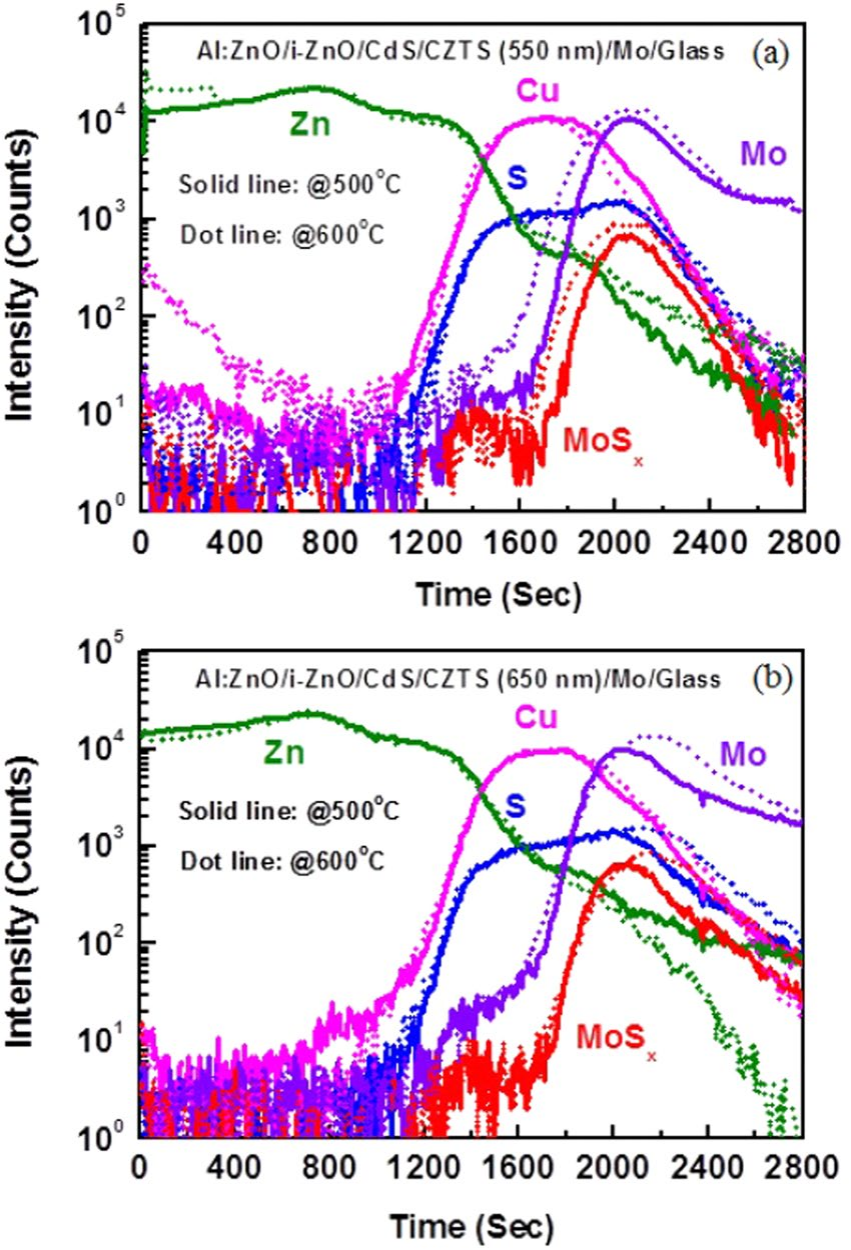
SIMS profile of elemental atoms from Al:ZnO/i-ZnO/CdS/CZTS/Mo/Glass structures with CZTS absorber layer thickness of (**a**) 550 nm and (**b**) 650 nm.

The elemental out-diffusion and formation of the interfacial layer are further investigated by time-of-flight secondary ion mass spectrometry (TOFSIMS) depth profiling analysis. For the depth profile analysis, CZTS was sputtered on Mo-coated glass using stoichiometric quaternary single target at RF power of 150 W and working pressure of 3.3 mTorr. Sulphur is present in the as-deposited CZTS film. Formation of thin interfacial MoS_x_ layer between Mo and CZTS was observed as indicated by a low MoS_x_ peak in Fig. 5(a). After sulphurization at 500 °C for 10 mins, the elemental out-diffusion of Mo into the CZTS layer was observed. The Mo-rich CZTS layer was formed after thermal treatment at high temperature. With the increase of annealing temperature, the thickness of MoS_x_ interfacial layer increases, as shown in Fig. 5(b–d). Elemental composition of Zn, Cu and Sn in the MoS_x_ layer also varied with temperature. SIMS analysis shows that annealing at the lower temperature (500 °C) results in higher amount of impurity (Cu, Sn and Zn) present in the MoS_x_ film. Significant reduction of the Zn, Cu and Sn composition was observed for post sulphurization at 600 °C. Since, elemental impurity atoms in MoS_x_ layer work as a trap centers, the reduction of these atoms thus improved solar cells performance through the reduction of series resistance and enhancement of shunt resistance of the devices. Furthermore, Mo and S content in the MoS_x_ layer significantly varies with sulphurization temperature, as shown in Fig. 6. Thus, it can be considered that after sulphurization, sputter-grown CZTS layer portrays three different regions, (i) CZTS layer, (ii) Mo-rich CZTS layer and (iii) MoS_x_ layer at CZTS/Mo interface. The formation of Mo-rich CZTS layer is due to the out-diffusion of Mo into the CZTS absorber layer. SIMS profiles of elemental atoms from the solar cell (Al:ZnO/i-ZnO/CdS/CZTS/Mo/Glass) with different thickness of CZTS absorber layer are given in Fig. 7. There is significant difference in the distribution of Mo and MoS_x_. For the device with CZTS thickness of 550 nm, presence of Mo-rich CZTS layer observed between MoS_x_ and CZTS layer. On the other hand, for the device with thicker CZTS layer, thickness of MoS_x_ layer increases and there is no trace of Mo-rich CZTS layer.

**Figure 8:**
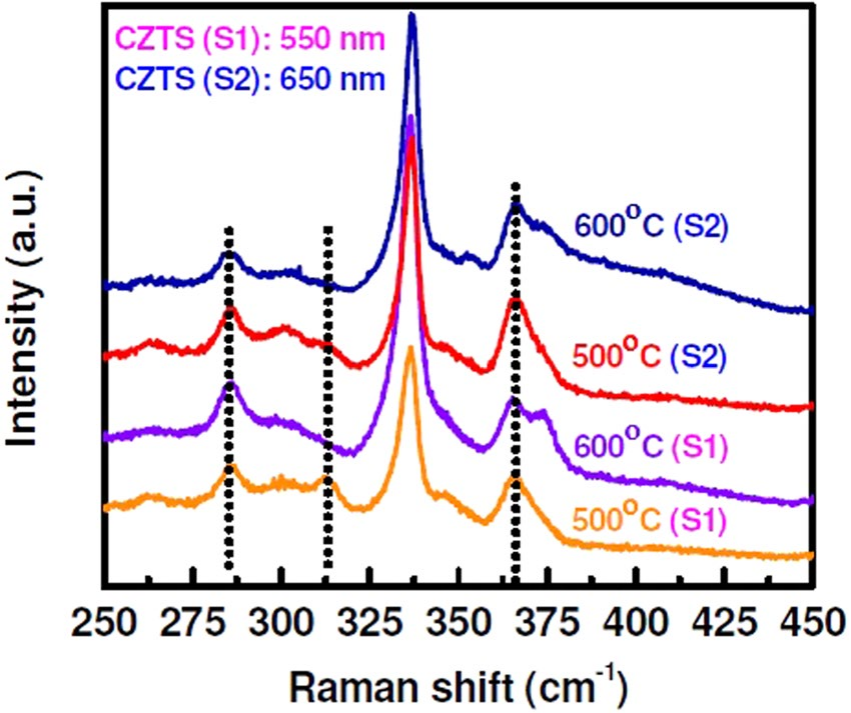
Raman spectra of sputter grown CZTS films on Mo-coated glass with different thickness (550 nm and 650 nm) after thermal treatment at 500 °C and 600 °C.

To investigate the interface layer quality we have further investigated Raman analysis. Figure 8 shows the Raman spectra of CZTS films. A strong peak is located at 336.5 cm^−1^ corresponding to main vibrational A1 symmetry mode from kesterite phase of CZTS^29, 32^. Peak at 285.6 cm^−1^ is related to SnS-like lattice vibration. Additional modes associated with secondary phase formation are also observed around 366–381 cm^−1^ became dominant in samples annealed at 600 °C. This mode is more prominent for the film with the CZTS thickness of 650 nm. These modes are associated with lattice vibrations close to the substrate with a MoS_x_-rich alloy formed at higher annealing temperature. Raman analysis renders that the thickness of MoS_x_ layer increases with CZTS layer thickness.

**Figure 9:**
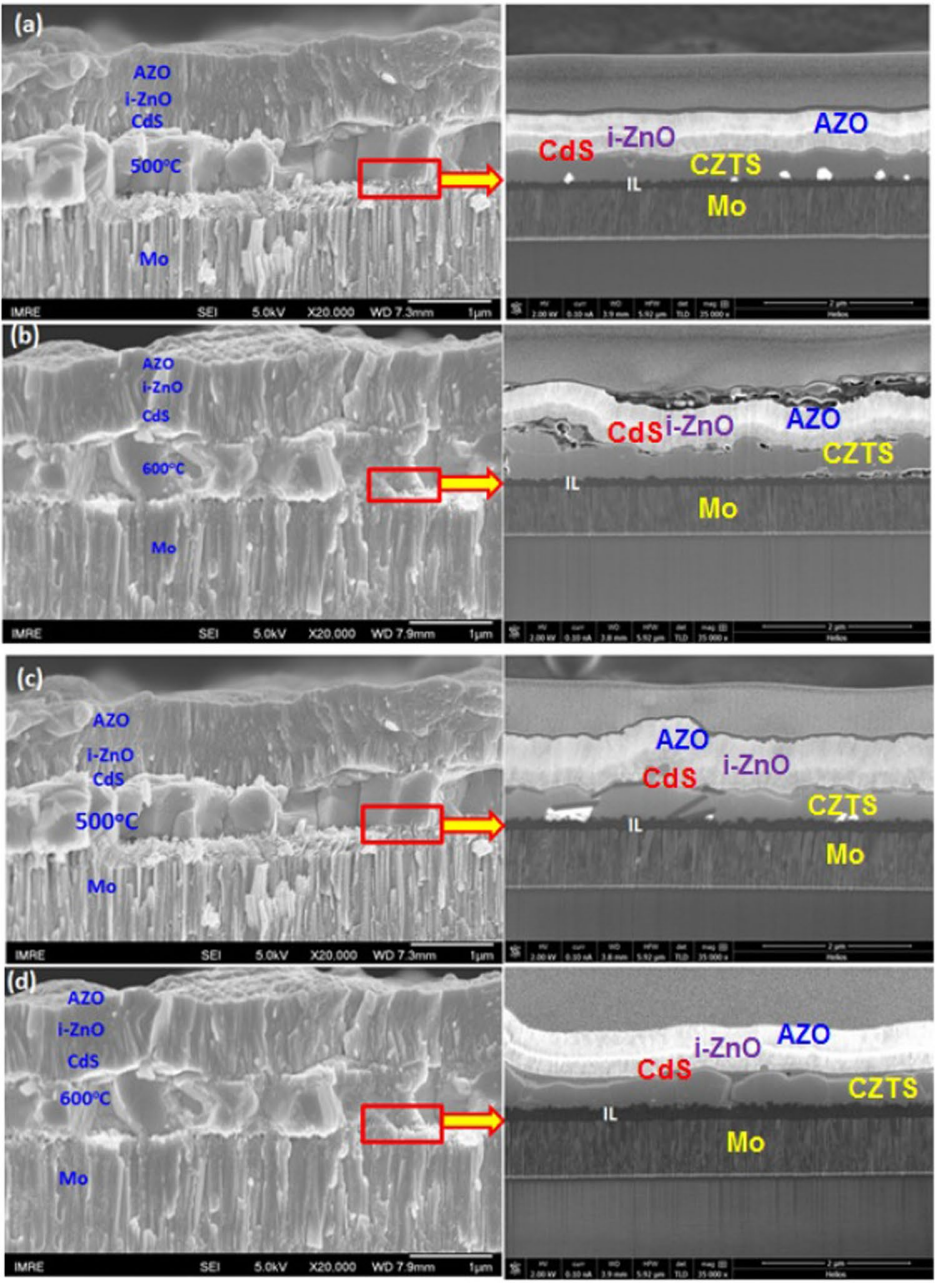
Cross-sectional SEM images of solar cell devices AZO/i-ZnO/CdS/CZTS/Mo/Glass. The CZTS layers (550 nm) sulphurized at (**a**) 500 °C and (**b**) 600 °C. The CZTS layers (650 nm) sulphurized at (**c**) 500 °C and (**d**) 600 °C. The respective cross section of the MoS_x_ interface layer (IL) obtained using focused ion beam milling (FIB) for each of the images in (**a–d**) as highlighted by the red box is shown to the right to determine the IL thickness. The thickness values have been tabulated in Table 2.

Formation of interfacial layer has been discussed by several groups. Scragg *et al*. investigated effects of back contact instability of Mo/CZTS solar cells^27^. They introduced titanium nitride (TiN)- as passivated back contact to suppress diffusion of sulphur atoms and reactions between CZTS and Mo, however, overall series resistance increases that results in reduction of fill factor. There are several approaches to improve the back contact between Mo and CZTS^11, 30, 33^. To reduce the series resistance of the CZTS/Mo device, ultrathin carbon layer was introduced on Mo-coated soda-lime glass (SLG) prior to the deposition of CZTS layer^11^. Ge *et al*. demonstrated CZTS based solar cells with transparent conducting oxides (TCO) as the front and back contacts to improve the efficiency of the CZTS based solar cells^33^. Formation of interfacial layer and elemental content are very crucial for the device performance. The SEM analysis was carried out to investigate the effect of sulphurization temperature on structural, morphological properties and interface layer formation. Cross-sectional SEM shown in Fig. 9 suggests that the grain size of the films sulphurized at different temperatures is almost similar. Further investigation on the elemental composition of the CZTS layer by line scan across the device (from glass to device) is measured by energy dispersive X-ray (EDX) analysis. The elemental distribution of CZTS films is significantly different when sulphurization temperature changes from 500 °C to 600 °C (Fig. S1). Elemental analysis shows that the Mo out-diffusion into the CZTS layer increases with the annealing temperatures. The thickness of MoS_x_ layer depends on annealing temperature and CZTS layer thickness.

The thickness of MoS_x_ layer is measured from high-resolution cross-sectional SEM image, as shown in Fig. 9, for the solar cells with CZTS layer annealed at 500 °C and 600 °C. The values are tabulated in Table 2. For the thicker device, even though the annealing temperature and duration are the same, the series resistance is much higher while the shunt resistance is lower than the solar cells with thinner CZTS layer (~550 nm). This is due to the presence of thick interfacial MoS_x_ (~240 nm) layer at the interface. Reduction of Zn content as well as increment of Cu and S content in the back contact region content was observed for the thicker device. This could cause formation of highly conductive Cu_2-x_S secondary phase providing shunt passes and, hence, reducing shunt resistance. Thickness of MoS_x_ layer, values of series resistance (R_s_) and shunt resistance (R_sh_) for a variety of sputter grown CZTS solar cells are given in Table 3. Sulphurization of sputter-grown CZTS layer at 600 °C has been beneficial to improve the charge transport property between CZTS and Mo through the reduction of series resistance^28^. On the other hand, reduction of impurity elements at the interface enhanced shunt resistance and thus lower the charge recombination^28^ and thus boost the efficiency of the device significantly (by 5 times) as compared to the devices fabricated using CZTS layer sulphurized at 500 °C. The presence of thick interface layer and absence of Mo-rich CZTS layer suppressed the overall performance of the device, even though absorber quality is improved after thermal treatment for the thicker device (CZTS thickness of 650 nm).

**Table 2:** Comparison of CZTS layer thickness and MoS_x_ layer thickness with annealing temperature.

CZTS layer thickness	Annealing temperature	MoS_x_ layer thickness
550 nm	500 °C	130 nm
	600 °C	142 nm
650 nm	500 °C	149 nm
	600 °C	240 nm

**Table 3:** Device characteristics of reported CZTS thin film solar cells made by sputtering.

Thickness of CZTS absorber layer (nm)	Growth process	Annealing condition	Thickness of MoS_x_ layer (nm)	R_s_, (Ohm.cm^2^)	R_sh_, (Ohm.cm^2^)	Ref.
1100	Sequential sputtering of precursor films	575 °C for 60 min	200	25.1	144.5	30
1000	Sequential sputtering of precursor films	560–575 °C for 60 min	300–400	18.5	819.2	37
1200	Sequential sputtering of precursor films	580 °C for 30 min	100	14.9	65	38
1270	Co-sputtering	500–525 °C for 3–4 hours	60	5.76	400	39
1000	Co-sputtering	260 °C for 75 min followed by annealing at 510 °C for 15 min	100	1.2	1183	40
1000	Sequential sputtering of precursor films	570 °C for 30 min	270	15.1	1630	41
1000	Sequential sputtering of precursor films	560 °C for 60 min	80	27.9	230.4	42
650	Single target sputtering	600 °C for 10 min	240	9.1	245	This study
550	Single target sputtering	600 °C for 10 min	142	5.9	401	This study

It is interesting to note that, even though the efficiency of the device is ~4.2% with CZTS layer thickness of ~550 nm, it is the highest reported value so far for the sputter-grown CZTS absorber layer with thickness ~500 nm. Fill-factor of the device is ~53%, which is relatively much lower as compared to the previously reported results^5, 34^. Thus, by increasing the FF of the device through suitable metal contact engineering, the efficiency of the solar cells can be increased further. Photovoltaic parameters of reported CZTS samples are tabulated in Table 4. Recently, Hou *et al*. reported CZTS based solar cells with the efficiency of 1.85% for low-temperature grown CZTS thin films of thickness ~110 nm^35^. *Su et al*. also showed that the Cd doped CZTS film exhibited excellent solar cell performance^36^. The replacement of Zn by Cd was considered the main cause to boost the efficiency^36^. Ge *et al*. also developed indium substituted CZTIS thin film absorber alloy and the novel bifacial device^33^. Similarly, the doping of Mo into CZTS layer can be considered to further design CZTS based solar cells to improve back contact quality. It is also worth to note that the sputter deposition method provides high quality thin film over large area with precise thickness. Sputter grown thin film has a huge potential for solar energy harvesting for a large scale deployment^45–49^. The structural quality, interfacial layer formation and chemical composition can be tuned *in-situ* during sputter deposition^50–53^. We believe that optimization of Mo content into the CZTS layer and control thickness of interfacial layer for thick CZTS layer can boost cell efficiency further.

**Table 4:** Photovoltaic parameters of reported sputter grown CZTS thin film solar cells.

CZTS thickness (nm)	Growth process	Annealing condition	J_sc_ (mA/cm^2^)	V_oc_ (mV)	FF (%)	Power conversion efficiency (%)	Ref.
1000	Sequential sputtering of precursor films	560–575 °C for 60 min	9.76	572	46.4	2.59	37
1200	Sequential sputtering of precursor films	580 °C for 30 min	22.5	492	34	3.8	38
1270	Co-sputtering	500–525 °C for 3–4 hours	19	603	55	6.2	39
1000	Co-sputtering	260 °C for 75 min followed by annealing at 510 °C for 15 min.	21.1	625	65.1	8.58	40
1000	Sequential sputtering of precursor films	570 °C for 30 min	15.97	641	42	4.3	41
750	Single target sputtering	570 °C for 60 min	19.17	513	52.7	5.2	43
1300	Co-sputtering	580 °C for 3 hours	17.9	610	62	6.77	44
650	Single target sputtering	600 °C for 10 min	14.4	608	50.2	4.4	This study
550	Single target sputtering	600 °C for 10 min	13.1	594	53.3	4.2	This study

## Conclusions

The temperature of sulphur treatment of sputter growth CZTS has the significant impact on the materials quality. Photo-generated carrier lifetime increases more than 3 times after suphurization at 600 °C. Mo out-diffusion and formation of Mo-rich CZTS are observed after sulphurization at 600 °C, and the two interfacial layers thus formed comprises of MoS_x_ and Mo-rich CZTS layer. The elemental composition at the CZTS-Mo interface and amount of Mo varies with different sulphurization temperature. The out-diffusion of Mo into CZTS layer plays an important role on the device performance. Series resistance remarkably decreases and shunt resistance increases after thermal treatment at 600 °C through the reduction of recombination centres at the interface. The overall efficiency of the solar cell improves nearly by five times when compared to CZTS-Mo interface sulphurized at 500 °C. The efficiency of ~4.2% with CZTS layer thickness of 550 nm is the highest achieved thus far for the sputter-grown CZTS absorber layer.

## Electronic supplementary material

Supplementary Information
