# Supplementary material for: Impact of molybdenum out diffusion and interface quality on the performance of sputter grown CZTS based solar cells
Source: Sci Rep. 2017 May 2;7:1350. doi: 10.1038/s41598-017-01605-7 (PMC5430996; doi:10.1038/s41598-017-01605-7)
Supplement: Supplementary file 1 — Supplementary Information [file 41598_2017_1605_MOESM1_ESM.docx]

**Impact of molybdenum out diffusion and interface quality on the performance of sputter grown CZTS based solar cells**

^1^Goutam Kumar Dalapati^*^, ^1,2^Siarhei Zhuk, ^1^SaeidMasudy-Panah, ^3^Ajay Kushwaha, ^1^Hwee Leng Seng, ^1^Vijila Chellappan, ^1^Vignesh Suresh, ^4^Zhenghua Su, ^5^Sudip Kumar Batabyal, ^1^Cheng Cheh Tan, ^4^Asim Guchhait, ^4,6^Lydia Helena Wong, ^2^Terence Kin Shun Wong, and ^1^Sudhiranjan Tripathy,

^1^Institute of Materials Research and Engineering, A*STAR (Agency for Science, Technology and Research), 2 Fusionopolis Way; Innovis, #08-03, Singapore 138634

^2^NOVITAS, School of Electrical and Electronic Engineering, Block S2, Nanyang Technological University, Nanyang Avenue, Singapore 639798

^3^Department of Metallurgy Engineering and Materials Science, Indian Institute of Technology Indore, Indore, MP, India 453552

^4^Energy Research Institute @ NTU, Nanyang Technological University, 50 Nanyang Drive, Research Techno Plaza, X-Frontier Block, Level 5, Singapore, 637553

^5^Amrita Centre for Industrial Research and Innovation (ACIRI), Amrita School of engineering, Coimbatore, Amrita University, Tamil Nadu, India-641112.

^6^School of Materials Science and Engineering, Nanyang Technological University, 50 Nanyang Avenue, Singapore 639798

Corresponding author:

*G. K. Dalapati: [dalapatig@imre.a-star.edu.sg](mailto:dalapatig@imre.a-star.edu.sg)

Further we have investigated on the elemental composition of the CZTS layer byline scan across the device (from glass to device), is measured by energy dispersive X-ray (EDX) analysis. The elemental distribution of CZTS films is significantly different when sulphurization temperature changes from 500°C to 600°C (Fig. S1). Elemental analysis shows that the Mo out-diffusion into the CZTS layer increases with the annealing temperatures. The thickness of MoS_x_ layer depends on annealing temperature and CZTS layer thickness.


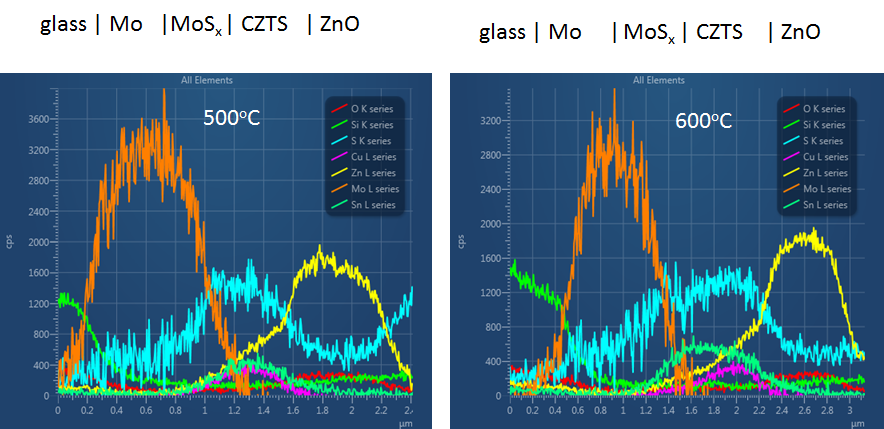


(a) (b)

Figure S1: Elemental distribution of atoms across the device. Thickness of CZTS layer is 550 nm and annealed at (a) 500°C and (b) 600°C.
